# From pronounced to imagined: improving speech decoding with multi-condition EEG data

**DOI:** 10.3389/fninf.2025.1583428

**Published:** 2025-06-27

**Authors:** Denise Alonso-Vázquez, Omar Mendoza-Montoya, Ricardo Caraza, Hector R. Martinez, Javier M. Antelis

**Affiliations:** ^1^Escuela de Ingeniería y Ciencias, Tecnologico de Monterrey, Monterrey, Mexico; ^2^Escuela de Medicina y Ciencias de la Salud, Tecnologico de Monterrey, Monterrey, Mexico

**Keywords:** imagined speech classification, EEG-based classification, overt speech, EEGNET, brain-computer interfaces

## Abstract

**Introduction:**

*Imagined speech* decoding using EEG holds promising applications for individuals with motor neuron diseases, although its performance remains limited due to small dataset sizes and the absence of sensory feedback. Here, we investigated whether incorporating EEG data from *overt* (pronounced) speech could enhance *imagined speech* classification.

**Methods:**

Our approach systematically compares four classification scenarios by modifying the training dataset: intra-subject (using only *imagined speech*, combining *overt* and *imagined speech*, and using only *overt speech*) and multi-subject (combining *overt speech* data from different participants with the *imagined speech* of the target participant). We implemented all scenarios using the convolutional neural network EEGNet. To this end, twenty-four healthy participants pronounced and imagined five Spanish words.

**Results:**

In binary word-pair classifications, combining *overt* and *imagined speech* data in the intra-subject scenario led to accuracy improvements of 3%–5.17% in four out of 10 word pairs, compared to training with *imagined speech* only. Although the highest individual accuracy (95%) was achieved with *imagined speech* alone, the inclusion of *overt speech* data allowed more participants to surpass 70% accuracy, increasing from 10 (*imagined only*) to 15 participants. In the intra-subject multi-class scenario, combining *overt* and *imagined speech* did not yield statistically significant improvements over using *imagined speech* exclusively.

**Discussion:**

Finally, we observed that features such as word length, phonological complexity, and frequency of use contributed to higher discriminability between certain *imagined* word pairs. These findings suggest that incorporating *overt speech* data can improve *imagined speech* decoding in individualized models, offering a feasible strategy to support the early adoption of brain-computer interfaces before speech deterioration occurs in individuals with motor neuron diseases.

## 1 Introduction

Speech is the primary mode of linguistic communication across all human cultures, defined as a complex system of articulated vocalizations (Fitch, 2010). Various diseases and medical conditions can lead to the progressive loss of speech, primarily due to upper and lower motor neuron degeneration and the deterioration of the muscles involved in speech production, even when some cognitive functions remain intact in many cases. Among these conditions are amyotrophic lateral sclerosis (ALS), primary lateral sclerosis (PLS), spinal-bulbar muscular atrophy (SBMA), motor aphasia in stroke, and pseudobulbar palsy (Tiryaki and Horak, 2014). This progressive degeneration of motor neurons can lead to a complete loss of the ability to be understood, profoundly affecting social interaction and emotional well-being. As a result, patients ultimately rely on assistive devices to facilitate communication (Eshghi et al., 2022).

To address the impact on communication due to motor difficulties present in these patients, speech and language therapists are commonly employed (Leigh et al., 2003). In addition, alternative and augmentative communication technologies, such as eye-tracking devices and brain-computer interfaces (BCIs), have been explored as potential solutions (Pugliese et al., 2022). Brain activity recording techniques include functional magnetic resonance imaging (fMRI), which offers excellent spatial resolution; electroencephalography (EEG), characterized by high temporal resolution; magnetoencephalography (MEG), which combines high temporal and spatial resolution; and electrocorticography (ECoG), an invasive method that provides both high temporal and spatial resolution (Zhao et al., 2023). Although MEG and ECoG have shown impressive performance in decoding neural representations of speech (Dash et al., 2020b; Moses et al., 2021; Proix et al., 2022), their high cost, limited accessibility, and invasive nature restrict their application in assistive communication technologies for everyday use. In contrast, EEG-based BCIs offer a non-invasive, cost-effective, and portable alternative, making them particularly promising for developing accessible communication systems (Lotte et al., 2007).

Several EEG-based BCIs have been developed using evoked potentials [e.g., P300, steady-state visual evoked potentials (SSVEPs)] or cognitive paradigms such as motor imagery (MI) (Aggarwal and Chugh, 2022). These approaches have enabled users to communicate by selecting predefined options, which can then be converted into text or auditory output. However, these paradigms, as well as eye-tracking systems, have limitations for direct communication through speech. While they facilitate indirect interaction, they do not directly translate neural representations of speech into a natural communication channel. As a result, there is a growing interest in developing EEG-based BCIs capable of decoding speech directly from brain signals, as this would enable intuitive, real-time communication and provide a more natural and direct alternative for individuals with severe speech impairments who are currently limited to indirect selection-based systems.

Speech decoding from brain signals has been extensively studied across different modalities, including *overt speech* (the most common form of verbal communication with audible volume and intonation), whispered speech (lower in volume and less distinct than normal speech), silent speech (articulated without sound), and imagined or covert speech (internally pronounced without vocalization or facial movement) (Nieto et al., 2022). Among these, *imagined speech* decoding has been proposed as a potential solution for individuals with severe motor impairments, as it does not require muscular engagement for communication. While this approach is particularly relevant for clinical populations, especially those affected by neurodegenerative diseases such as amyotrophic lateral sclerosis (ALS), only a few studies have applied speech decoding techniques in these contexts. For example, Dash et al. (2020a) demonstrated the feasibility of decoding spoken and imagined phrases in ALS patients using MEG. More recently, Angrick et al. (2024) developed a real-time speech synthesis system based on ECoG signals from a chronically implanted ALS patient, achieving intelligible output while preserving the speaker voice profile. Although this study relied on overt speech, it represents an important step toward applications in more advanced stages of ALS, where imagined speech decoding may become necessary. In a related line of work, Dash et al. (2024) used imagined and overt speech tasks with MEG to distinguish ALS patients from healthy controls based on spectral and functional connectivity features. However, this study focused on classifying clinical condition rather than decoding the semantic content of imagined speech.

Most studies in this field, however, have been conducted on young, healthy participants, namely individuals without diagnosed speech disorders, using non-invasive EEG recordings (Lee et al., 2020; Vorontsova et al., 2021; Datta and Boulgouris, 2021; Sarmiento et al., 2021; Hossain et al., 2024). This participant selection is largely influenced by the challenges inherent to EEG data collection, particularly in *imagined speech* tasks. Unlike other BCI paradigms, *imagined speech* lacks immediate sensory feedback, making it difficult for participants to assess whether they are correctly performing the task. Additionally, the extended duration required for data collection, which arises from the necessity of multiple repetitions and the cognitive effort involved, further limits the feasibility of large-scale studies (Combrisson and Jerbi, 2015). These constraints are exacerbated in clinical populations, where factors such as fatigue and cognitive load must also be considered. In the field of *imagined speech* word decoding, this translates to a reduced dataset for training classification models. Therefore, it is important to identify strategies that improve class differentiation when classifying *imagined speech* words, even with limited data, to subsequently develop methods that can be implemented online and allow us to decode words in patients.

In light of this, and considering that motor neuron loss is a progressive process, meaning that patients retain the ability to speak in the early stages of the disease, we explored different ways of using EEG signals from *overt speech* to train *imagined speech* models. Previous studies have analyzed similarities between different neural processes of speech in relation to *imagined speech*, such as perceived speech (the hearing of spoken words) (Moon et al., 2022) and visual imagery (mentally picturing a scene or object in the mind without external stimuli) (Lee et al., 2020). It has been shown that overt and *imagined speech* share certain temporal and spatial characteristics (Nieto et al., 2022; Lee et al., 2019; Martin et al., 2014). *Overt speech* has also been employed to train *imagined speech* models using common spatial patterns (CSP) alongside traditional classifiers (Rekrut et al., 2022), or through a convolutional autoencoder to transfer features from *overt speech* EEG to *imagined speech* classification (Lee et al., 2023).

To investigate how the combination of these two neural speech processes (*overt speech* and *imagined speech*) affects *imagined speech* classification, we designed and conducted an experiment in which 24 healthy participants pronounced and imagined five Spanish words used in assistive devices for individuals with speech limitations. The words varied in connotation, number of syllables, frequency of use, grammatical class, semantic meaning, and functional role within a sentence. We performed an event-related potential (ERP) analysis for each condition: *overt speech* and *imagined speech*. For classification, we used EEGNet, a convolutional neural network designed for EEG-based brain-computer interfaces (Lawhern et al., 2018). These methods were evaluated in four classification scenarios designed to explore how *overt speech* data could contribute to *imagined speech* classification: three intra-subject scenarios (where the model is trained and tested within the same participant) and one multi-subject scenario (where the model is trained using data from multiple participants combined with a portion of data from the target participant, and tested on the remaining data from that same participant). Each scenario involved different combinations of *overt* and *imagined speech* data during training. Within each scenario, we performed three classification tasks: (i) binary word vs. word, (ii) binary short vs. long words, and (iii) five-class multi-class classification (all words).

Throughout this work, we address various research questions aimed at gaining deeper insight into the brain responses associated with *overt* and *imagined speech* tasks. Specifically, we pose the following research questions (RQs):

- RQ1. In which temporal window and under which ERP components do overt and *imagined speech* responses show the greatest similarity? Moreover, how can this information guide the selection of the analysis window for subsequent classification tasks?
- RQ2. Does including *overt speech* data during model training significantly improve the classification of *imagined speech* words?
- RQ3. Which data grouping strategy during model training achieves the highest accuracy and robustness in *imagined speech* word classification?
- RQ4. Based on RQ2, does this behavior hold in both binary and multi-class classification scenarios?
- RQ5. Which characteristics or features (e.g., phonetic, semantic, or articulatory complexity) facilitate the discrimination of certain pairs of *imagined speech* words?

These questions guide our study and enable a systematic evaluation of how combining both conditions impacts the classification of *imagined speech* words. Through the analysis in RQ1, we identify patterns of similarity between overt and *imagined speech* responses, informing the selection of the analysis window. Subsequently, via RQ2, we explore the effect of incorporating *overt speech* data in model training and, with RQ4, verify whether this effect persists across different classification scenarios. In RQ3, we evaluate various data grouping strategies to optimize classification accuracy and robustness. Finally, in RQ5, we analyze which linguistic characteristics facilitate word discrimination in the *imagined speech* task.

## 2 Materials and methods

The methods for data acquisition and preparation, analysis, as well as the classification methods, are described below.

### 2.1 Participants

The experiment was conducted with 24 healthy participants with an age range of 20–47 years (mean = 24, std = 6 years), 15 men and nine women. All participants were native Spanish speakers, with normal or corrected vision, right-handed, with no diagnosed neurological conditions or speech disorders. Before the start of the experiment, all participants provided written informed consent allowing the use of their data, including audio and video files. The experimental protocol was designed following the principles of the Declaration of Helsinki and approved by the Institutional Research Ethics Committee of the Instituto Tecnológico y de Estudios Superiores de Monterrey under the ruling EHE-2023-9a.

### 2.2 Experimental protocol

**Figure 1:**
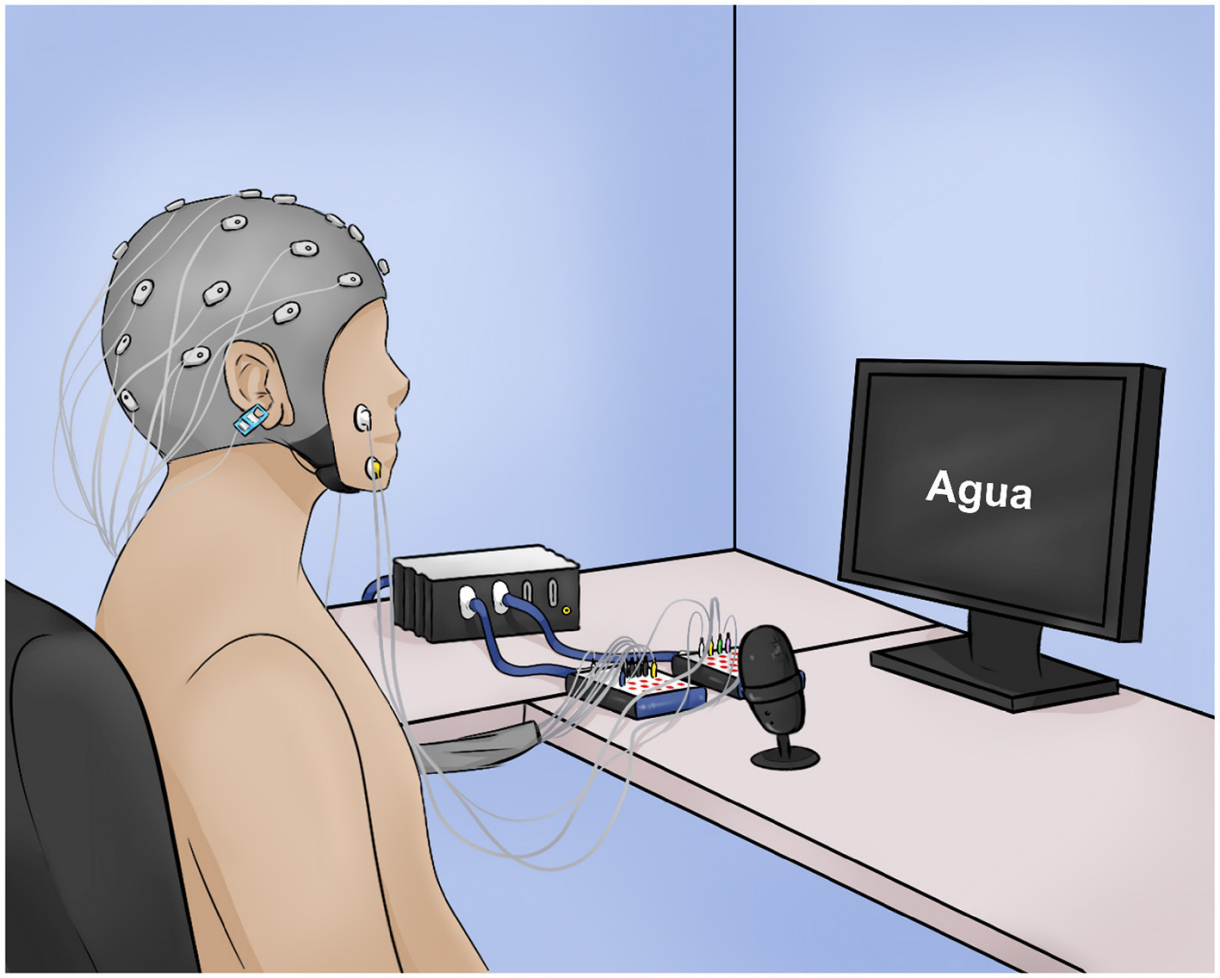
Illustration of the experimental setup. The participant is seated in front of the monitor with the EEG cap, the EMG electrodes, and the microphone. The monitor shows the word “agua,” (“water”) corresponding to the task stage.

The experiment involved overt and imagined pronunciation (representing the two conditions: *overt speech* and *imagined speech*) of five Spanish words: “si,” “no,” “agua,” “comida,” and “dormir,” which translate to “yes,” “no,” “water,” “food,” and “sleep,” respectively. This set of words was selected because they are basic for communication, especially for individuals with motor and language limitations. Additionally, these words vary in aspects such as semantic meaning, grammatical class, connotation, and number of syllables, which enriches the set of words and contributes to the study of certain characteristics of speech production. Moreover, the *overt speech* data were previously used in another study for word decoding in different classification scenarios (Alonso-Vázquez et al., 2023b). The recordings were conducted in a single session. The participant sat in front of an 18.5-inch monitor, wore a cap with EEG electrodes, and had two electromyography electrodes placed on each side of the face, one on the major zygomatic muscle and the other on the triangular muscle, while audio was recorded with a microphone (see Figure 1). In this work, only the EEG data will be analyzed.

**Figure 2:**
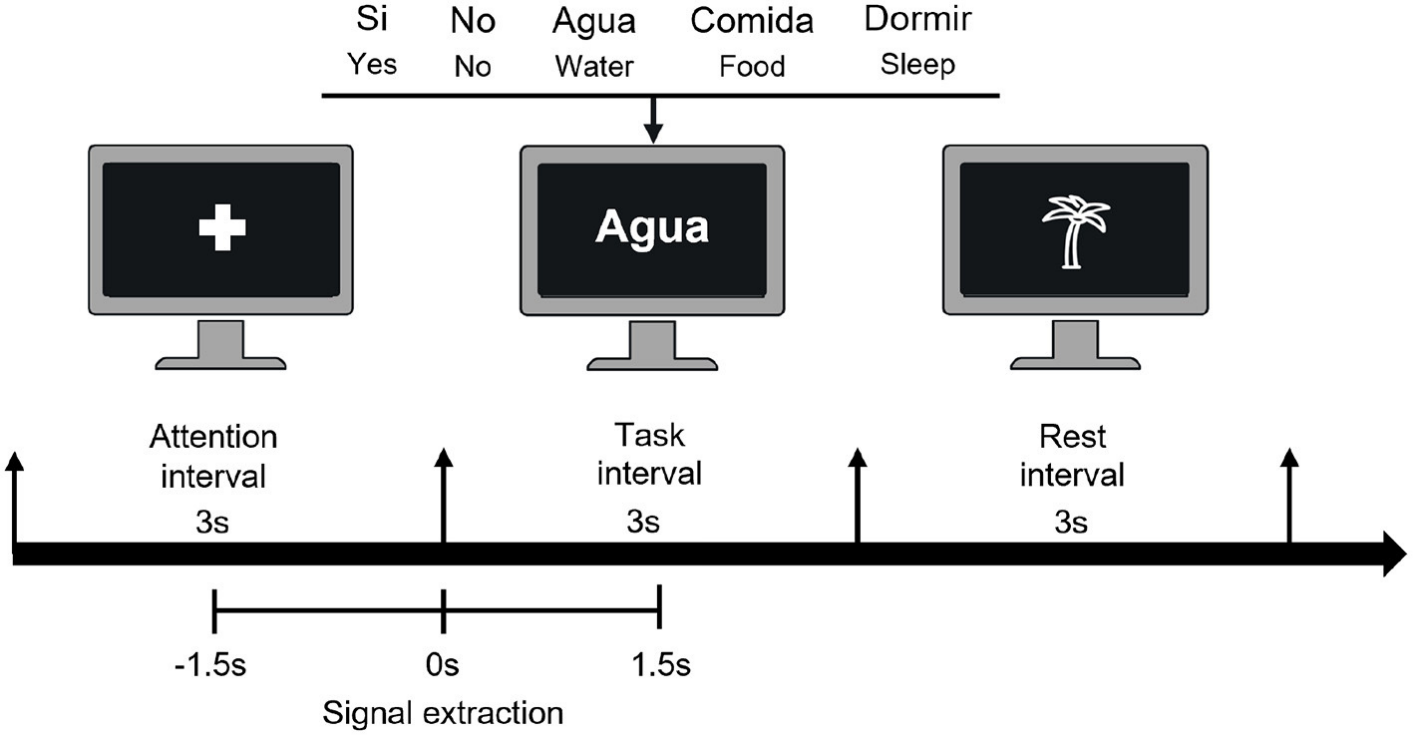
Timeline illustration of trial events. The trial begins with a 3-s attention period marked by a fixation cross. Following this, the participant is presented with one of the five words for 3 s, which they must pronounce only once (either *imagined* or *overt speech*). Finally, there is a 3-s rest period (palm). Additionally, the lower section of the image displays the data segment from –1.5 to 1.5s, which represents the time window of interest.

Before starting the experiment, the participants were briefed on the two conditions, *overt speech* was defined as the natural pronunciation of the word at a normal volume and pitch. While *imagined speech* involved pronouncing the word in their mind without making any sound or gesturing any movement. The experiment was conducted in eight experimental blocks, four for each experimental condition. The order of the blocks was random. At the beginning of each block, participants were duly instructed whether the task would involve *overt* or *imagined speech*. A graphical user interface (GUI) displayed on the monitor guided the experiment by showing visual information. A trial (see Figure 2) was divided into three intervals of 3 s each: attention, task, and rest. In the attention interval, the instruction was to avoid movements and stay attentive by looking at the fixation cross. Three seconds later, one of the five words was randomly shown on the screen, and the participant had to pronounce it once, either as *imagined speech* or *overt speech* according to the type of block as instructed to the participant. Finally, during the rest interval, participants were advised to rest and blink, avoid sudden movements, and wait for the next fixation cross. Eight blocks of 50 trials each were recorded, totaling 200 trials for each condition and 40 trials for each word in each condition. Between the end and the beginning of each block, the participant rested for ~3 min.

### 2.3 Data recording

EEG signals from 32 active electrodes (Ag/AgCl) were recorded, uniformly distributed over the scalp according to the 10/10 standard. The ground electrode was placed at the AFz position and the reference on the right earlobe. For recording and digitizing the signals, the g.HIAMP bio-signal amplifier (g.tec) was used. The data were recorded at a sampling rate of 1,200 Hz, applying a Butterworth band-pass filter from 0.5 to 500 Hz and a Notch filter at 60Hz.

### 2.4 Data processing and preparation

The data were trimmed from –1.5s to 1.5 s relative to the stimulus onset (see Figure 2), were downsampled to 256Hz, and had an eighth-order Butterworth digital filter applied from 1 to 30Hz to reduce most of the muscle-related activity (Goncharova et al., 2003). Noisy channels were removed, eliminating between 0 and 4 channels per participant. Independent Component Analysis (ICA) was used as a method to eliminate artifacts from the EEG signals. First, we decomposed the data into 32 independent components. Then, we visually identified the components associated with cardiac, ocular, and motor artifacts–the latter mainly present during *overt speech*. Components were considered artifactual if they exhibited characteristic spatial patterns (e.g., frontal for eye blinks, central for heartbeat, temporal for EMG) and irregular high-frequency activity in the time domain, particularly in the case of muscle artifacts. We selected between 1 and 6 components per participant as artifacts. Finally, we reconstructed the EEG signal excluding these selected components. To ensure the quality of the EEG data, we implemented a process for detecting noisy trials by calculating the probability distribution functions of the peak-to-peak voltages and their standard deviations across all channels. This allowed us to determine appropriate thresholds for identifying noisy trials. A trial was considered noisy and excluded from further analysis if, in at least one of the electrodes, the peak-to-peak voltage exceeded 150 μV and the standard deviation surpassed 20 μV. As a result of this preprocessing, the data structure for each subject has the following form: $\text{x}\in {\mathbb{R}}^{\left(N_{Channels}\cdot N_{samples}\cdot N_{trials}\right)}$, where *N*_*Channels*_ = 32, *N*_*samples*_ = 769, *N*_*trials*_ = 200 for *overt* and *imagined speech* in the ideal case in a subject where no channels or trials were eliminated.

### 2.5 EEG analysis

To explore the temporal window in which *overt speech* and *imagined speech* responses show the greatest similarity, and to gain a deeper understanding of the underlying patterns in the EEG signals (RQ1), we conducted various analyses. Specifically, we examined the temporal dynamics of brain activity during task execution by calculating event-related potentials (ERPs). The data were filtered from 4 to 20Hz using a Butterworth band-pass filter. We applied baseline correction to each epoch using the pre-stimulus period from –400 to –100 ms. The ERPs were calculated by averaging the pre- and post-stimulus brain activity of all epochs for each of the channels and participants. The grand average was obtained by averaging the ERPs of all participants in each of the channels. In the calculation of the ERPs, averaging the responses at the individual level before calculating a group average ensures that each participant contributes proportionally to the final outcome, respecting individual variability and the dataset size of each participant (Luck and Kappenman, 2013).

For each ERP, significant positive and negative peaks were computed using a statistical test based on the non-parametric kernel density estimation (KDE) method (Chen, 2017). Here, the probability density function (PDF) of the ERP in the pre-stimulus interval, specifically from –0.2 to 0 s where no *overt* or *imagined speech* is performed, is estimated with KDE method. Then, it is tested according to a confidence level α = 0.01 whether each amplitude value of the ERP belongs to the left or right tails of the estimated PDF, which represent ERP negative or positive values significantly different from the ERP in the pre-stimulus interval, respectively. This procedure was performed separately for each channel and for each condition.

### 2.6 Classification scenarios

**Figure 3:**
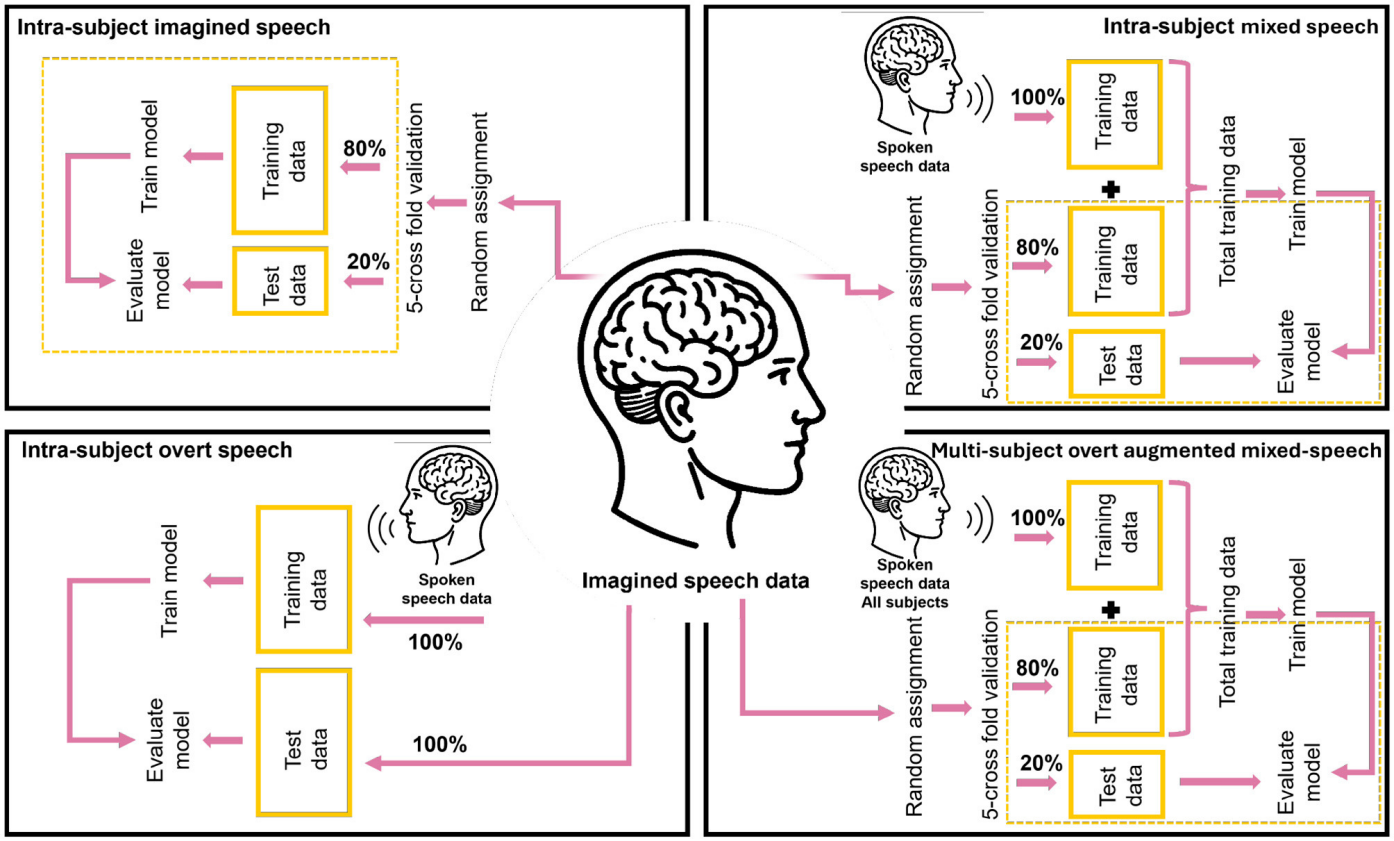
Classification scenarios. Each block shows the distribution of *imagined speech* and spoken speech data for training and testing, according to each of the intra-subject scenarios and the multi-subject scenario.

To compare different training strategies for imagined speech decoding, we defined three intra-subject scenarios and one multi-subject scenario, which incorporates data from other participants. These four configurations were designed to examine the impact of incorporating *overt speech* data into the model training for classifying *imagined speech* words (RQ2), as well as to evaluate which data grouping strategy yields the highest accuracy and robustness (RQ3). The scenarios are illustrated in Figure 3 and in Table 1.

- Intra-subject scenarios. The training and evaluation of the model are performed exclusively with data from the same participant.

1. Intra-subject *imagined speech* training: The model is trained and tested using only *imagined speech* data, with 80% used for training and 20% for testing via cross-validation.
2. Intra-subject mixed speech training: The model is trained with both *overt* and *imagined speech* data (100 and 80% respectively), and tested using 20% of the *imagined speech* data via cross-validation.
3. Intra-subject *overt speech* training: The model is trained exclusively with *overt speech* data and tested with *imagined speech* data.

- Multi-subject *overt* augmented mixed-speech training: The model is trained using 80% of the *imagined speech* data from the target participant, augmented with 100% of the *overt speech* data from all participants, and tested with the remaining *imagined speech* data from the target participant via cross-validation. This configuration mirrors the intra-subject mixed speech training, with the addition of overt data from other participants to assess whether it improves classification performance for the target individual.

**Table 1:** Summary of the training scenarios evaluated in this study.

**Scenario**	**Subject level**	**Training data**	**Test data**
1. Intra-subject imagined speech training	Intra-subject	Imagined speech from the target participant (80%)	Imagined speech from the target participant (20%)
2. Intra-subject mixed speech training	Intra-subject	Imagined (80%) + overt (100%) speech from the target participant	Imagined speech from the target participant (20%)
3. Intra-subject overt speech training	Intra-subject	Overt speech from the target participant (100%)	Imagined speech from the target participant (100%)
4. Multi-subject overt augmented mixed-speech training	Multi-subject	Imagined speech (80%) from the target participant + overt speech (100%) from all other participants	Imagined speech from the target participant (20%)

All evaluations were subject-dependent, using only imagined speech data for testing.

All scenarios described above were subject-dependent: in all cases, the test data came exclusively from the same participant whose data were partially included in the training set.

To explore whether the effect of including *overt speech* data during training holds in both binary and multiclass classification scenarios (RQ4), we evaluated two settings within each configuration. *Word vs. word*: to determine which word pairs are most effectively discriminated and to examine how linguistic properties may influence classification (RQ5), we performed ten binary classifications covering all possible pairwise combinations among the five words. *All words*: In this setting, we study the recognition among the five words, which corresponds to a five-class multiclass classification. For participants without any removed trials, each class consisted of 40 trials.

### 2.7 Classification method: EEGNet

EEGNet is a compact convolutional neural network architecture designed for EEG-based brain-computer interfaces (BCIs). It is adaptable to various BCI paradigms and can be trained with a minimal amount of data, capturing temporal and spatial patterns in EEG signals (Lawhern et al., 2018).

#### 2.7.1 Network architecture and hyperparameters

The time segment used for classification was from 0 to 0.5s for each epoch, which represents 129 samples. Thus, the input matrix to the network has the following form: $\text{x}\in {\mathbb{R}}^{\left(N_{Channels}\cdot N_{samples}\right)}$, where *N*_*Channels*_ = 32, and *N*_*samples*_ = 129, for *overt* and *imagined speech* in the ideal case in a subject where no channels were eliminated. The time window selected for classification (from 0 to 0.5 s after stimulus onset) was defined based on a prior analysis of event-related potentials (ERPs), as described in Section 3.1. This analysis identified an interval in which both conditions (overt and imagined speech) exhibited comparable brain dynamics. This allowed us to select a segment in which similarities between conditions were maximized, with the aim of using overt speech data to train models for classifying imagined speech.

**Figure 4:**
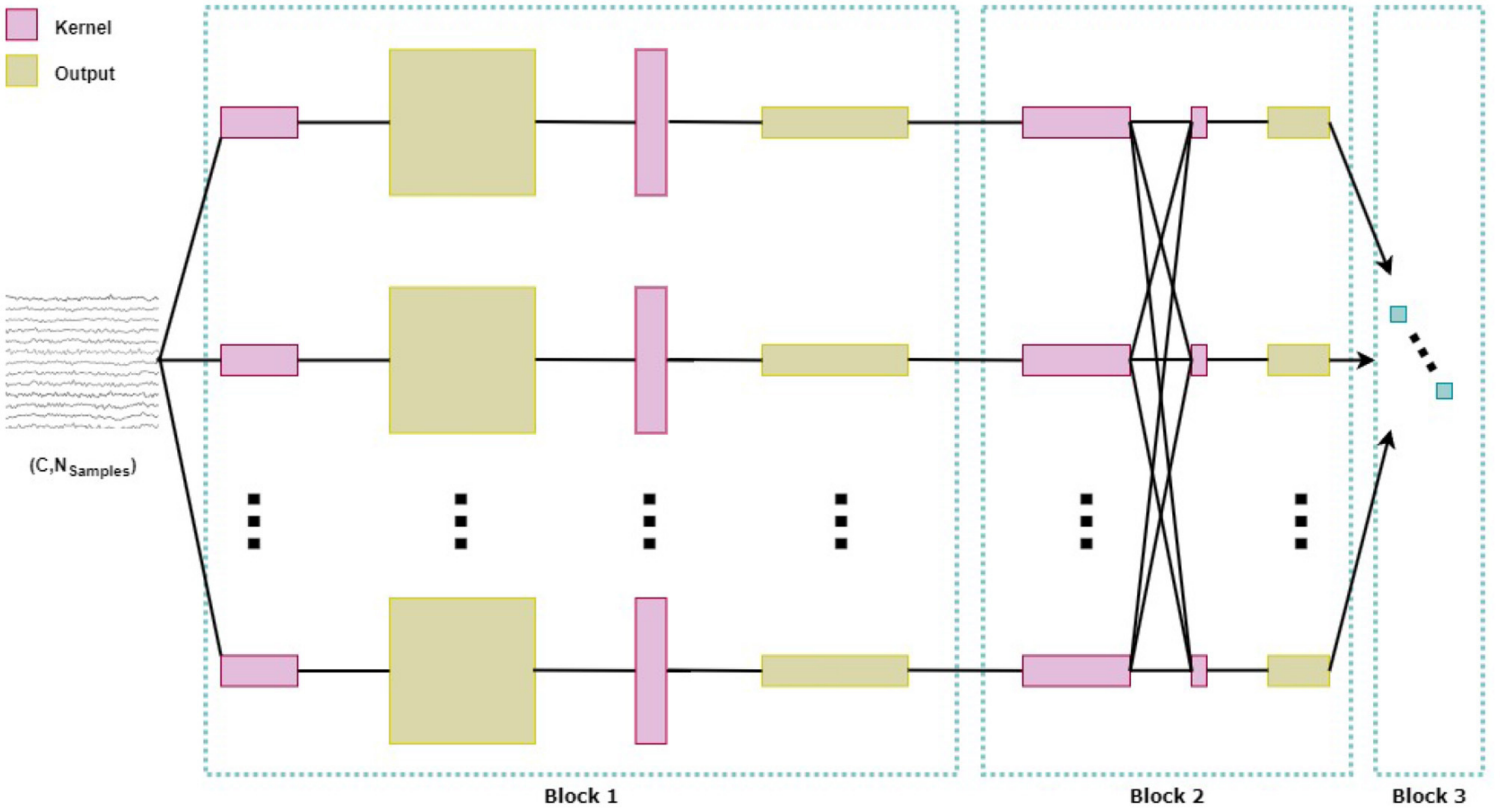
Architecture of the convolutional neural network EEGNet.

Figure 4 shows the architecture of EEGNet, which consists of three main blocks: The first block begins with a sequence of two convolutional stages. The first stage performs a temporal convolution to learn frequency filters. Different parameters were used in this stage depending on the classification scenario (see Supplementary material). The parameter *F*1 is the number of temporal filters with a kernel size of (1, 128), corresponding to half of the sampling rate. The second stage of the first block involves a depthwise convolution that learns spatial filters specific to each temporal filter, with a size of (*C*, 1), where *C* is the number of channels, being *C* = 32 for participants where all channels were retained. The depth parameter *D* indicates the number of spatial filters to be learned within each temporal convolution, set to different values depending on the classification scenario (see Supplementary material). This combination of temporal and spatial filtering is inspired by the Filter Bank Common Spatial Pattern (FBCSP) algorithm (Ang et al., 2008). FBCSP addresses the limitations of the Common Spatial Pattern (CSP) algorithm, whose effectiveness depends on the EEG's operational frequency band. Batch normalization is then applied before using the Exponential Linear Unit (ELU) activation function and a dropout layer to regularize the model, with a probability of 0.65. The block concludes with an average pooling layer of size (1, 4) to reduce the sampling rate of the signal to 64 Hz.

The second block consists of a separable convolution that combines a depthwise convolution of size (1, 16) with a pointwise convolution. In this case, *F*2 = *F*1*D, where *F*2 is the number of pointwise filters to be learned. As in the first block, batch normalization is applied, followed by ELU activation and a dropout layer with a probability of 0.65 for regularization. This block also includes an average pooling layer of size (1, 8) to reduce the dimensions. Finally, the third block is responsible for classification using the softmax function. Additional information about the network can be found in Lawhern et al. (2018).

### 2.8 Validation procedure and performance metrics

In the first two intra-subject scenarios (*imagined speech* and mixed speech training) and the multi-subject overt augmented mixed-speech training scenario, EEGNet was trained and tested using five-fold cross-validation on *imagined speech* data. The *imagined speech* dataset was divided into five groups. Four of these groups, along with the entire set of spoken speech data (depending on the scenario), were used to train the model, while the remaining group was used for evaluation. This process was repeated five times, ensuring that the training and testing datasets were always mutually exclusive. During each iteration, two metrics were calculated: overall accuracy and recall per class. Overall accuracy represents the fraction of test instances correctly classified (Japkowicz and Shah, 2011). It is defined as follows:


(1) $$\begin{array}{l} {\text{Acc}}_{T}\left(f\right)=\frac{1}{\left|T\right|}\overset{\left|T\right|}{\underset{i=1}{\sum }}I\left(f\left(x_{i}\right)=y_{i}\right), \end{array}$$

where |*T*| denotes the total number of samples in the test dataset *T*. The indicator function *I*(*a*) outputs 1 if the predicted class *a* is correct and 0 otherwise, *f*(*x*_*i*_) represents the label predicted by the model *f* for the *i*-th sample, while *y*_*i*_ is the actual label for that sample. This metric indicates the quality of the classification but is heavily affected by imbalanced classes, favoring the majority class. To provide information on class-specific performance, recall per class is proposed. Recall is defined as the number of correctly classified elements of a class divided by the total number of elements that should have been classified in that class, i.e., performance of the model in correctly identifying positive cases (Foody, 2023). This is defined as follows:


(2) $$\begin{array}{l} Recall_{class_{c}}=\frac{\sum_{i=1}^{\left|T\right|}I\left(y_{i}=c\wedge f\left(x_{i}\right)=c\right)}{\sum_{i=1}^{\left|T\right|}I\left(y_{i}=c\right)} \end{array}$$

where c is the specific class for which the recall or precision is computed.

In the multi-subject overt augmented mixed-speech training scenario, data from different subjects were combined, resulting in different voltage ranges depending on the subject. These variations in voltage ranges are due to individual variability in brain activity and factors such as electrode placement and scalp conductivity, which can vary between subjects. Similarly, data from for *overt* and *imagined speech* showed differences in voltage ranges for some subjects. To ensure uniform contribution from all features and maintain a consistent scale for each specific channel across epochs, we applied a *z*-score normalization. This process standardized the data so that each channel had a mean of zero and a standard deviation of one. For spoken speech data, standardization was performed only once, as this set remained constant during cross-validation. However, for *imagined speech* data, the parameters (mean and standard deviation) were calculated on the training set for each cross-validation fold, and these same parameters were then used to transform the test set in each iteration.

#### 2.8.1 Statistical significance of classification

In the classification of small datasets, as is often the case with brain signals, it is necessary to consider that the chance level depends on the number of available data points, and the theoretical behavior (e.g., a 50% chance level for two classes) only holds in large sample sets (Combrisson and Jerbi, 2015). To calculate the statistical significance thresholds for binary classification *word vs. word* and multiclass classification (five classes), we assume that classification errors follow a cumulative binomial distribution, where for *n* samples and *c* classes, the probability of correctly predicting the class at least *z* times by chance is given by:


(3) P(z)=∑i=znni(1c)i(c-1c)n-i

Therefore, using the Matlab function X = binoinv(Y, n, P), which returns the smallest integer X such that the binomial cumulative distribution function evaluated at X is equal to or exceeds Y, we calculated the statistical significance threshold with α = 0.05. The parameters used were Y = 1-α, which is the cumulative probability complement of the significance level, *n* is the sample size, i.e., the number of observations (80 for two classes and 200 for five classes), and P is the probability of success in each trial, i.e., 1/c. By multiplying X by 100/n, we converted this number of successes into a percentage of the total sample size. The thresholds we obtained were 58.75% for binary *word vs. word* classification and 24.50% for multiclass classification. In addition to the analytical estimation, we empirically validated the chance levels using a bootstrapping approach. Specifically, for each classification case, we permuted the class labels of the training set 500 times and trained a new model on each permuted dataset using the same architecture and parameters. The classification accuracy was then evaluated on the true test labels, building a null distribution of accuracies under random labeling. The 95th percentile of this distribution was used as an empirical threshold to determine statistical significance. This empirical bootstrapping analysis yielded thresholds of 64.10% for binary word vs. word classification, and 26.05% for multiclass classification. These empirically derived thresholds are more conservative than the analytically estimated ones, and will therefore be used throughout the manuscript to assess statistical significance.

Additionally, we performed the Wilcoxon signed-rank test (Wilcoxon, 1945), which is a non-parametric test used to compare two samples or to compare a sample with a specific value. It evaluates whether the medians of the distributions are significantly different from each other.

To evaluate the significance of the results, we use the Wilcoxon signed-rank test to compare our results to the baselines. The test was conducted with a significance level of α = 0.01 and compared the distributions of the results against the calculated statistical significance threshold for each case.

## 3 Results

The results found in both the analysis of the EEG signals and the classification experiments are shown below.

### 3.1 EEG analysis

**Figure 5:**
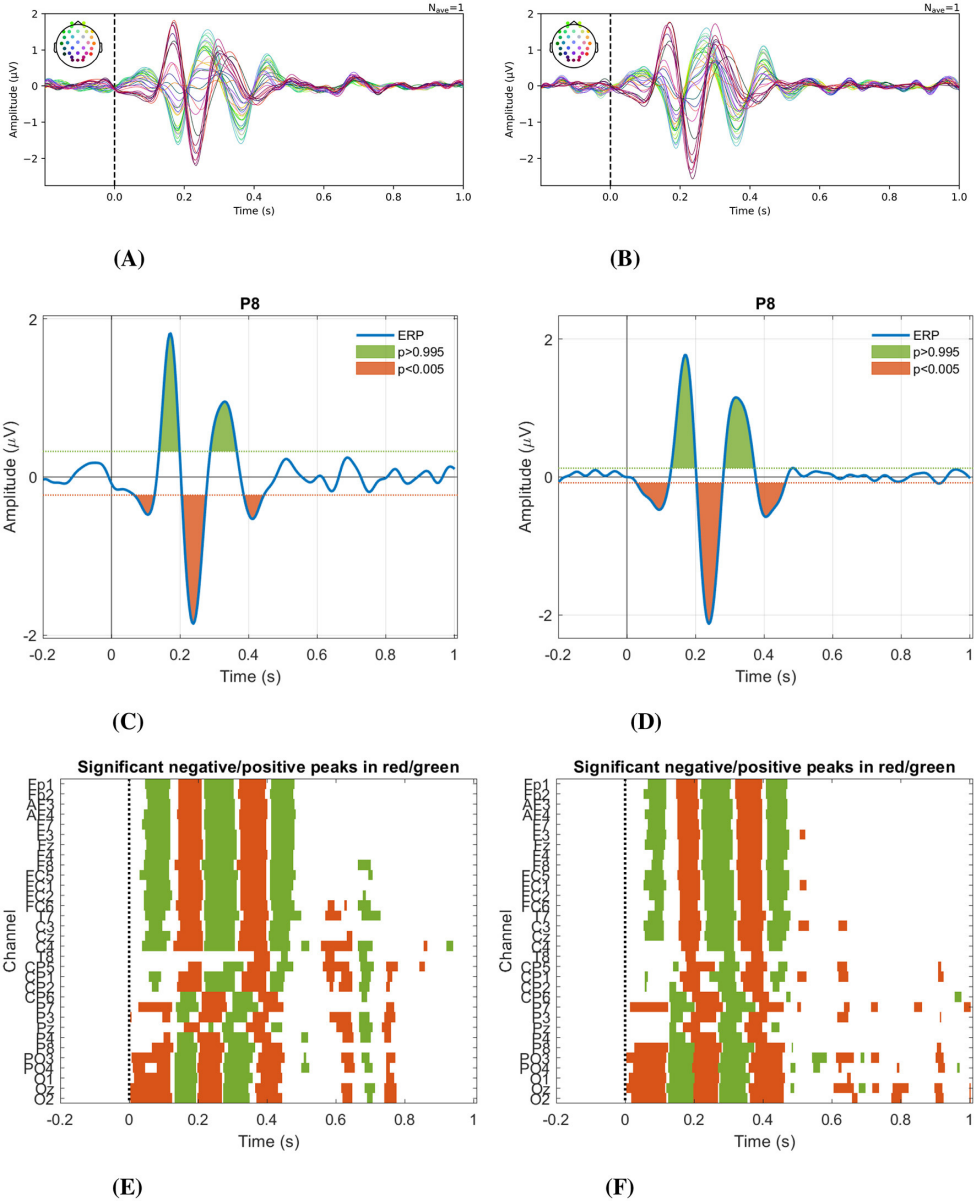
Event-related potentials and the statistical significance of the peaks. ERPs calculated across all participants in all channels for **(A)** *overt speech*, and **(B)** *imagined speech*. Representative statistically significant peaks (P8 channel) α = 0.01, color green for positive peaks and color orange for negative peaks, **(C)** *overt speech*, and **(D)** *imagined speech*. Distribution of significant peaks (α = 0.01) over time across the 32 channel **(E)** *overt speech*, and **(F)** *imagined speech*.

Figures 5A, B show the ERPs calculated across all participants in each condition. These potentials indicate a typical brain response to the presented visual stimuli. In both conditions, we observed a latency difference of ~50 ms between parietal/occipital and frontal channels. This difference suggests a sequential activation pattern, where information is initially processed in posterior sensory regions and subsequently transferred to anterior areas associated with higher-order linguistic and cognitive functions. This observation is consistent with cascade processing models described in previous studies on visual word recognition (Hauk et al., 2006) and rapid categorization of visual stimuli (Thorpe et al., 1996).

In Figures 5C, D, statistically significant peaks in the P8 channel of each task can be seen, while Figures 5E, F illustrate the distribution of significant peaks over time across the 32 channels. A similar behavior regarding the spatial and temporal distribution of significant peaks in the evoked response between *overt* and *imagined speech* is observed up to 500 ms, where after this time in the *overt* condition, significant peaks are concentrated between 600ms and 800ms.

The waveform of these ERPs and their statistical analysis reveal the possible presence of five components. The first corresponds to the P100, which is a positive peak found around 100 ms and is related to basic visual processing. The second component is negative, close to 200 ms, corresponding to the N200, characterized by a prolonged negativity between 100 and 300ms after the stimulus, especially over frontocentral areas (Schmitt et al., 2000). The third is a positive peak between 250 and 300 ms, known as P300. This component corresponds to the unpredictable response to any stimulus (Van Petten and Luka, 2012). The fourth component is a negative peak between 330ms and 410ms, known as N400, which reflects the additional work the brain must do to process an unrelated word to the active concept when the word is presented (Swaab et al., 2012). The fifth is a positive peak observed around 450ms, mainly in electrodes located in frontal and frontocentral areas of the brain, corresponding to the P450, a component related to cognitive inhibition (Mercado et al., 2013).

Observing the period of time in the ERPs where the brain response is similar during both tasks and verifying the statistical significance of the observed peaks determined the time segment taken for classification. This is because the data from the two conditions will be mixed, and it is sought to be during the period of time in which they have the greatest similarity. Although the early ERP components include visual and cognitive responses, this time window also encompasses the onset of linguistic processing. Therefore, it is not only the segment where both modalities are comparable, but also the period where the brain begins to encode speech-related information–making it a reliable window for classification. Previous work has shown that this early interval also contains semantic and speech-related information, both in *imagined speech* classification tasks (Alonso-Vázquez et al., 2023a) and in comparisons against non-linguistic visual stimuli such as fixation crosses (Alonso-Vázquez et al., 2023b).

### 3.2 Classification

Below are the results of the bi-class and multiclass classification under the different classification scenarios.

#### 3.2.1 Word vs. word

**Figure 6:**
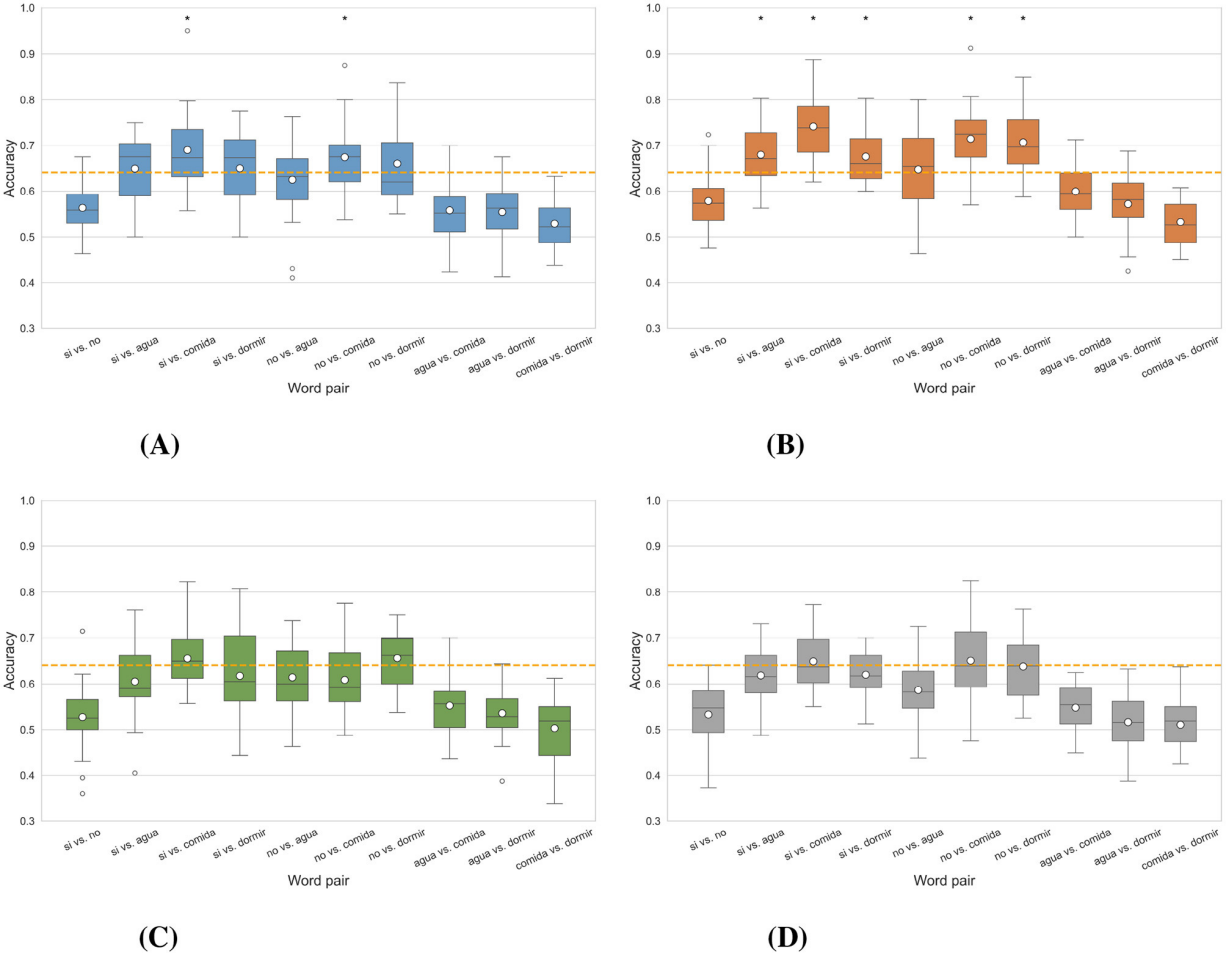
Distribution of classification accuracy for each word pair obtained in the different scenarios in the case *word vs. word*. The asterisk (*) indicates the word pair in which the results are statistically significant, i.e., they exceed the statistical significance threshold (Wilcoxon signed-rank test, *p* < 0.05), marked by the dashed line located at 64.10%. **(A)** Intra-subject imagined speech training. **(B)** Intra-subject mixed speech training. **(C)** Intra-subject overt speech training. **(D)** Multi-subject overt augmented mixed-speech training.

In Figure 6, boxplots illustrate the accuracies obtained in each of the four binary *Word vs. word* classification scenarios for all participants. The scenarios that had word pairs exceeding the statistical significance threshold of 64.10% Wilcoxon signed-rank test (*p* < 0.05) were the intra-subject *imagined speech* training and the intra-subject mixed speech training scenarios (see Figures 6A, B), with the first having two word pairs above the threshold and the second having five. In intra-subject *overt speech* training (Figure 6C) and the multi-subject overt augmented mixed-speech training scenario (Figure 6D), no word pairs exceeded the threshold.

**Figure 7:**
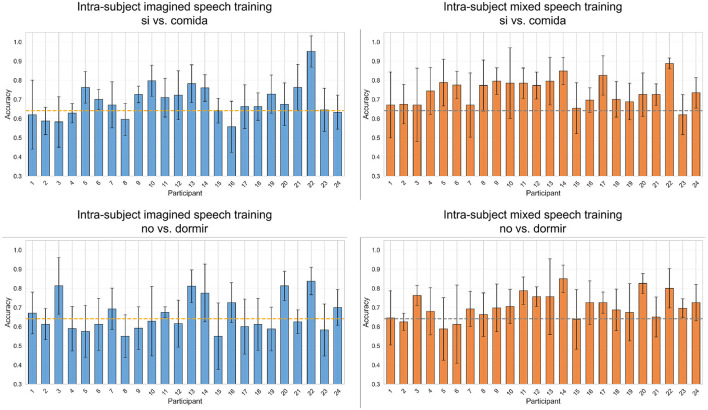
Classification accuracies per participant for the word pairs “si” vs. “comida” and “no” vs. “dormir” using intra-subject imagined speech training and intra-subject mixed speech training. The error bar represents the standard deviation obtained by averaging values from five-fold cross-validation. The statistical significance threshold is indicated by the dashed line located at 64.10%.

The pairs “si” vs. “comida,” and “no” vs. “dormir,” were the only ones that exceeded the statistical significance threshold in two scenarios. In contrast, the pairs “si” vs. “no,” “agua” vs. “comida,” “agua” vs. “dormir,” and “comida” vs. “dormir,” did not reach this threshold in any scenario. Figure 7 shows the classification results per participant for the word pairs “si” vs. “comida” and “no” vs. “dormir,” considering the first two intra-subject scenarios (*imagined speech* training and mixed speech training). Each graph displays the average accuracy obtained through cross-validation, along with its error bars, for each of the 24 participants. In the intra-subject *imagined speech* training scenario (left panels), for “si” vs. “comida,” 16 participants exceed the statistical significance threshold. Notably, participant 22 achieves the highest accuracy (95%). Regarding those who surpass 70% accuracy, there are 10 in “si” vs. “comida” and 6 in “no” vs. “dormir.” Additionally, for “no” vs. “dormir,” ten participants exceed the significance threshold, while the rest present accuracy values below it. In the intra-subject mixed speech training scenario (right panels), only one participant falls below the significance threshold in the pair “si” vs. “comida,” and four in the pair “no” vs. “dormir.” Participant 22 again stands out with the highest accuracy, reaching 88.75% in “si” vs. “comida.” In this scenario, 15 participants exceed 70% accuracy in “si” vs. “comida” and 11 do so in “no” vs. “dormir.” Overall, the intra-subject mixed speech training scenario shows superior performance, with a greater number of participants surpassing 70% accuracy in both tasks. The Supplementary material contains the participant-by-participant results for each of the remaining word pairs.

The intra-subject mixed speech training scenario recorded, on average, the highest accuracies per word pair: “si” vs. “comida,” reached 74.16% compared to 68.99% in the intra-subject *imagined speech* training scenario; “no” vs. “comida,” 71.46% vs. 67.40%; “no” vs. “dormir,” 70.69% vs. 66.02%; “si” vs. “agua,” 67.94% vs. 64.92%; “si” vs. “dormir,” 67.53% vs. 65.00%; and “no” vs. “agua” 64.74% vs. 62.53%. According to the Wilcoxon signed-rank test (*p* < 0.05), these differences were statistically significant in four of the five pairs that exceeded the significance threshold, highlighting the superiority of the intra-subject mixed speech training scenario in “si” vs. “agua,” “si” vs. “comida,” “no” vs. “comida,” and “no” vs. “dormir.”

**Figure 8:**
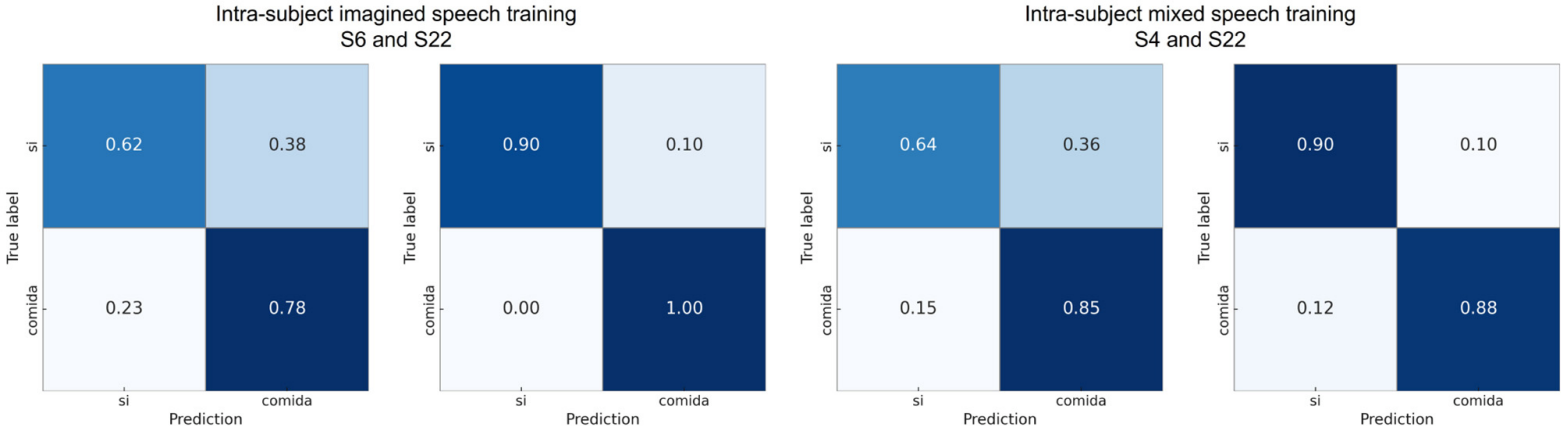
Confusion matrices using intra-subject imagined speech training and intra-subject mixed speech training for the word pair “si” vs. “comida,” showing results from participants with performance close to the average and from the participant with the highest accuracy.

Figure 8 shows the confusion matrices calculated with one participant close to the average in each scenario and with the best-performing participant in the pair “sí” vs. “comida.” It can be observed that in the intra-subject mixed speech training scenario for participant 22, the classes are balanced, while in the other confusion matrices there is a slight tendency toward better recognition of the word “comida.” Finally, to verify that the intra-subject mixed speech training scenario does not exhibit bias toward either of the two classes, Supplementary Figure S2 shows the recall for both classes in each word pair. The statistical analysis (Wilcoxon signed-rank, *p* < 0.05) revealed no significant differences between the recall values of the two classes in any of the pairs, suggesting balanced performance under this scenario.

In addition to the scenarios included in the main analysis, two additional configurations were explored: (5) Multi-subject imagined augmented imagined-speech training: training using only imagined speech data from all participants. (6) Multi-subject mixed augmented mixed-speech training: training using all types of speech data (imagined and overt) from all participants. However, in both cases, the model showed difficulties in training effectively. Even when using the best-performing participant (22) and the most discriminable word pair (“si” vs. “comida”), the test accuracy remained unstable and did not show a clear improvement trend over the epochs. For this reason, these scenarios were not included in the comparative analysis, but representative training and validation accuracy curves can be found in the Supplementary Figures S3, S4.

#### 3.2.2 Short words vs. long words

As a consequence of the findings reported in Section 3.2.1, where one-syllable words were classified with better performance compared to multi-syllable words, the classification of *short words vs. long words* was proposed. The short words group includes “si” and “no” (one syllable), while the long words group contains “agua” and “dormir” (two syllables). Each group consists of 80 trials (40 per word). The results of this classification were computed under the intra-subject *imagined speech* training and intra-subject mixed speech training scenarios, which performed best in Section 3.2.1. In all cases (accuracy and recall per class), the statistical significance level of 58.29% (95th percentile of the permutation test, α=0.05) was surpassed. Additionally, the distribution of accuracy in the intra-subject mixed speech training scenario is higher than in the intra-subject *imagined speech* training scenario.

The average accuracy in the intra-subject *imagined speech* training scenario was 67.58%, whereas in the intra-subject mixed speech training scenario it reached 70.80%. According to the Wilcoxon signed-rank test (*p* < 0.05), this difference is statistically significant, indicating that the intra-subject mixed speech training scenario achieved superior performance in the binary classification of short words vs. long words. Additionally, the recalls of both classes were evaluated in the intra-subject mixed speech training scenario, and once again, the Wilcoxon signed-rank test (*p* < 0.05) revealed no significant differences, indicating the absence of bias toward either class.

**Figure 9:**
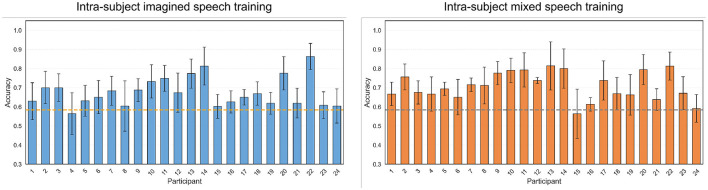
Classification accuracies per participant for *short word vs. long word* using intra-subject imagined speech training and intra-subject mixed speech training. The error bar represents the standard deviation obtained by averaging the values from five-fold cross-validation. The statistical significance threshold is indicated by the dashed line located at 58.29%.

Figure 9 shows the classification accuracy values per participant for the intra-subject *imagined speech* training and intra-subject mixed speech training scenarios. In both cases, only one participant fails to reach the statistical significance threshold. Moreover, the previously noted trend is confirmed: in the intra-subject *imagined speech* training scenario 6 participants exceed 70% accuracy, while in the intra-subject mixed speech training scenario, 12 participants do so. Likewise, the participant with the highest accuracy was found in the intra-subject *imagined speech* training scenario (86.30%), coinciding with the same individual who obtained the best score in the previous section.

**Figure 10:**
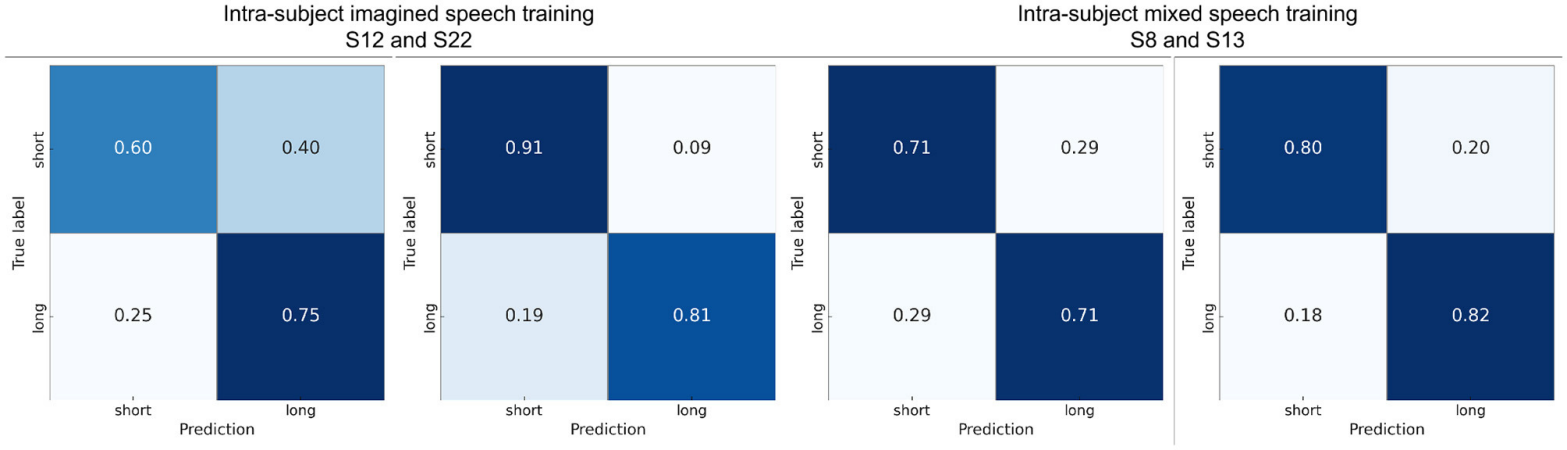
Confusion matrices using intra-subject imagined speech training and intra-subject mixed speech training for short words vs. long words showing results from participants with performance close to the average and from the participant with the highest accuracy.

The confusion matrices reveal that (see Figure 10) although both classes were generally well distinguished, errors tended to occur more frequently in short words being misclassified as long words. This suggests that longer words might elicit more distinctive EEG patterns, facilitating their correct classification.

#### 3.2.3 All words

**Figure 11:**
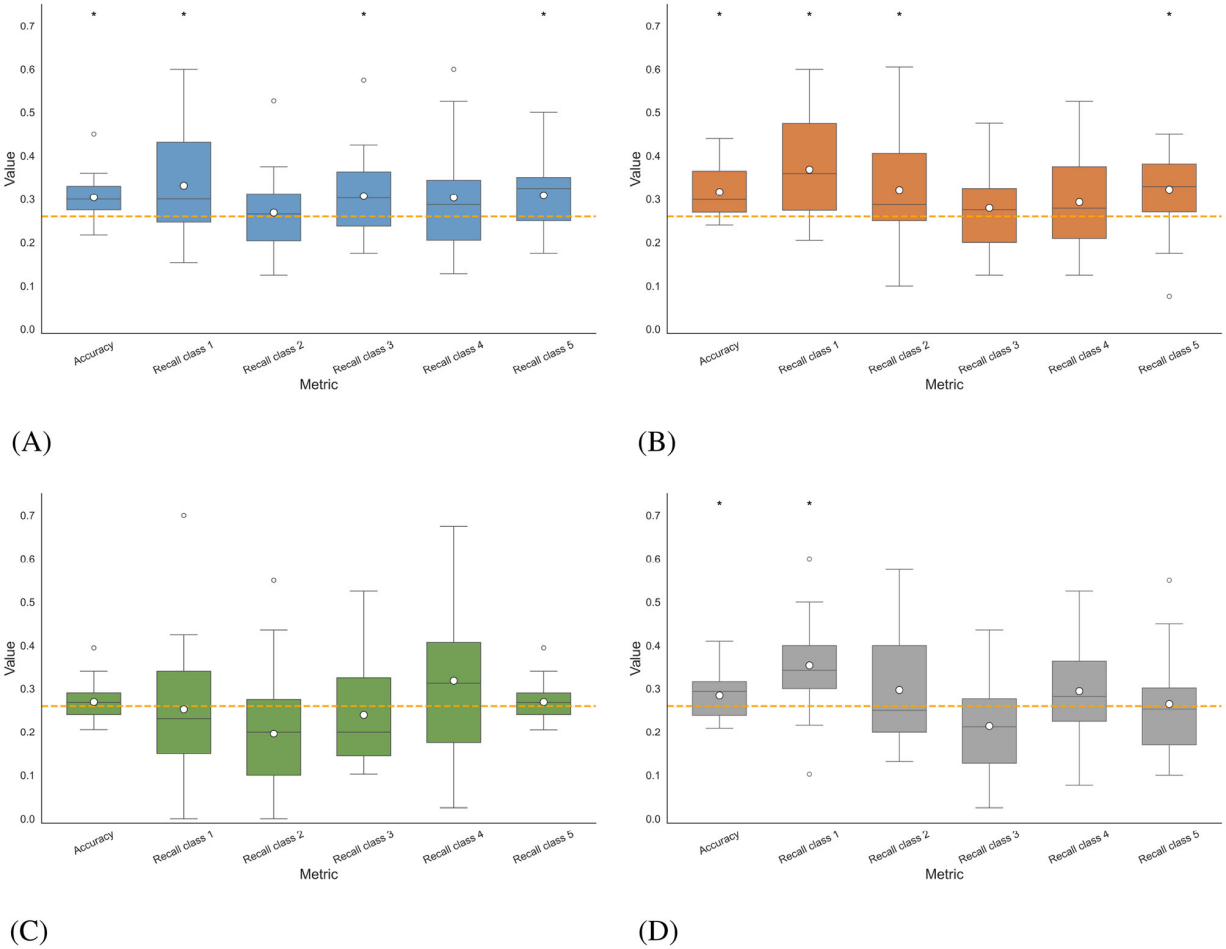
Distribution of the average accuracy and recall by class for multiclass classification obtained in the different scenarios. The asterisk (*) indicates the metric for which the results are statistically significant, meaning they exceed the statistical significance threshold for 5 classes set at 26.05% (Wilcoxon signed-rank test, *p* < 0.05). The white dot in the center of each boxplot represents the mean. **(A)** Intra-subject imagined speech training. **(B)** Intra-subject mixed speech training. **(C)** Intra-subject overt speech training. **(D)** Multi-subject overt augmented mixed-speech training.

**Figure 12:**
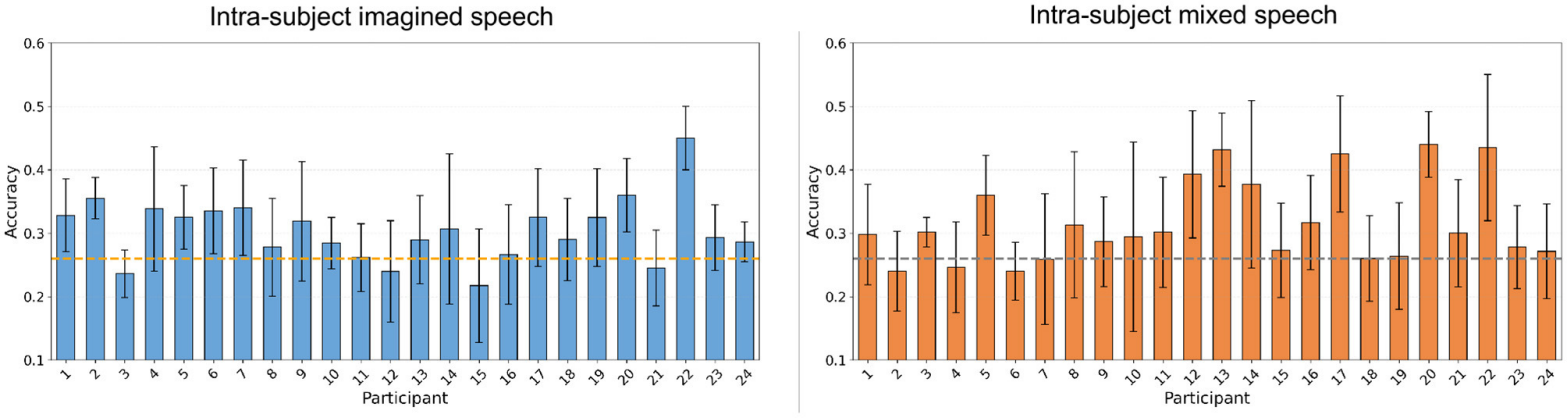
Classification accuracies per participant in the multi-class classification task, using intra-subject imagined speech training and intra-subject mixed speech training. The error bar represents the standard deviation obtained by averaging the values from 5-fold cross-validation. The statistical significance threshold is indicated by the dashed line located at 26.05%.

In Figure 11, the accuracies obtained for all participants in the four multi-class classification scenarios, as well as the recalls corresponding to each class, are illustrated using boxplots. In intra-subject *imagined speech* training and intra-subject mixed speech training scenarios, and in the multi-subject overt augmented mixed-speech training scenario, the accuracy exceeded the statistical significance threshold with *p* < 0.05 (Wilcoxon signed-rank test), set at 26.05% for a five-class problem. The best results were observed in the intra-subject mixed speech training scenario (Figure 11C), with an average accuracy of 31.68%, followed by the intra-subject *imagined speech* training scenario (Figure 11A) with 30.40%. In contrast, the intra-subject *overt speech* training scenario and the multi-subject overt augmented mixed-speech training scenario, (see Figures 11C, D) reached values of 26.95% and 28.47%, respectively. According to the Wilcoxon signed-rank test, the comparison between the intra-subject *imagined speech* training and intra-subject mixed speech training scenarios yielded no statistically significant differences (*p*>0.05). However, both scenarios showed significant differences when compared to the intra-subject *overt speech* training scenario (*p* < 0.05). Class 1, corresponding to the word “si,” was the only one whose recall exceeded the statistical significance threshold in three out of the four scenarios, indicating that the proportion of true positives identified by the model (relative to all actual positives) surpasses that benchmark. It was followed by class 5 corresponding to the word “dormir,” where the threshold was exceeded in two out of the four scenarios. The class with the lowest performance in identifying true positives was the one corresponding to the word “comida.” The participant-specific results, illustrated in Figure 12, indicate that, although the differences between the intra-subject *imagined speech* training and intra-subject mixed speech training scenarios were not statistically significant (*p*>0.05, Wilcoxon signed-rank test), the trend observed in previous sections persists: the highest accuracy (45%) is recorded in the intra-subject *imagined speech* training scenario and again corresponds to participant 22. However, the intra-subject mixed speech training scenario shows a greater number of participants (4) exceeding 40% accuracy, compared to just one participant in the intra-subject *imagined speech* training scenario. Furthermore, in in the latter, 3 participants do not reach the significance threshold, while in the former, 2 participants fail to do so.

**Figure 13:**
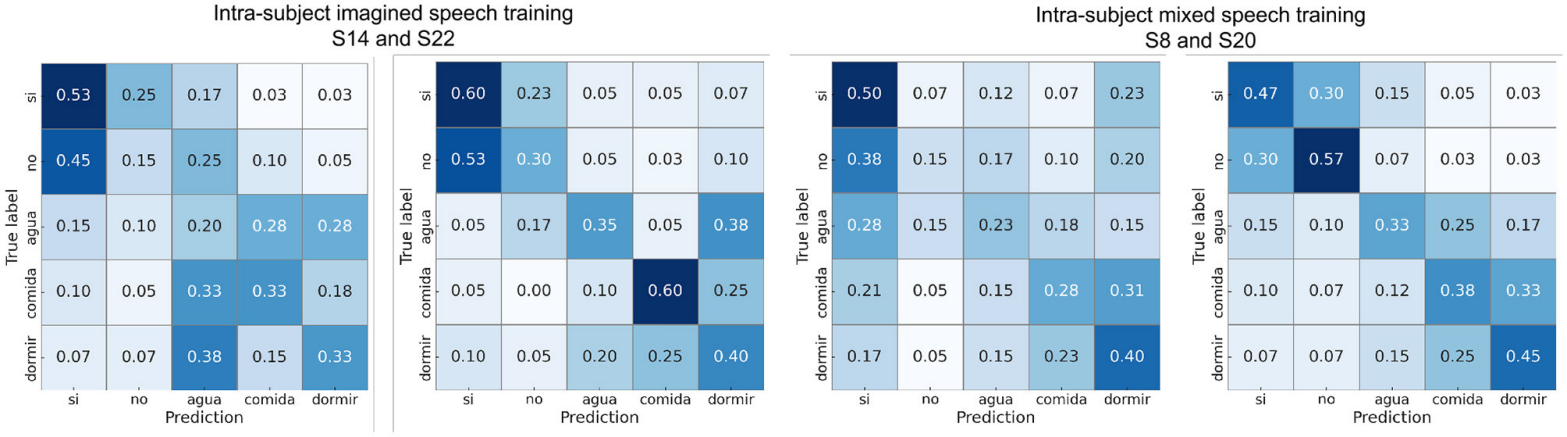
Confusion matrices using intra-subject imagined speech training and intra-subject mixed speech training in the multiclass classification showing results from participants with performance close to the average and from the participant with the highest accuracy.

Figure 13 presents the confusion matrices calculated for one participant near the mean accuracy and for the best-performing participant in each scenario. For almost all subjects, the word “si” exhibited the highest proportion of correct predictions (diagonal values). Overall, the word “agua” was the most frequently confused with other words.

## 4 Discussion

In this study, we explored different ways of incorporating EEG signals recorded during *overt speech* tasks to enhance the decoding of *imagined speech* words. Initially, a time-domain analysis was carried out by computing ERPs for each condition and identifying statistically significant peaks. Our results indicate that, during the first 500 ms following stimulus presentation, the spatial and temporal distribution of these peaks is very similar for both *overt* and *imagined speech*. In particular, a sequential activation pattern was observed, characterized by an initial response in parietal and occipital regions followed by activation in frontal areas, consistent with cascade models of visual and linguistic processing (Hauk et al., 2006; Thorpe et al., 1996). This similarity, combined with the presence of early semantic and cognitive ERP components, enabled us to define a temporal interval in which both conditions exhibit a comparable evolution while capturing meaningful linguistic representations.

To assess the usefulness of *overt speech* data for *imagined speech* classification, we proposed four classification scenarios (three intra-subject and one multi-subject), each involving different combinations of data in the training set. Likewise, a trend was observed in individual participant behavior: in the word pairs where the intra-subject mixed speech training scenario performed better, most participants exceeded the statistical significance threshold, and the number of participants above 70% accuracy was higher compared with the intra-subject *imagined speech* training scenario. Although a higher individual accuracy was observed in the intra-subject *imagined speech* training scenario, the broader success across participants in the intra-subject mixed speech training scenario suggests that the classification method used in the latter may be more robust or better adapted to the individual characteristics. Nevertheless, the consistently high performance of participant 22 in both scenarios illustrates inter-subject variability and suggests that certain individuals exhibit more distinguishable brain activity patterns for the words studied Samek et al. (2013).

In two out of the four scenarios, the word pairs “si” vs. “comida” and “no” vs. “dormir” surpassed the statistical significance threshold. These words differ in length, phonetic complexity, grammatical class, type of concept, semantic meaning, and frequency of use. The words “si” and “no” are very short (one syllable), whereas “comida” (three syllables) and “dormir” (two syllables) have a more extensive phonetic structure. These differences may give rise to more contrasting brain activity patterns when pronouncing or imagining the words. Furthermore, “si” and “no” are frequently used and serve grammatical functions (affirmation/negation), while “comida” is a noun and “dormir” is a verb, evoking different semantic networks (objects vs. actions). Meanwhile, pairs such as “si” vs. “no,” “agua” vs. “comida,” “agua” vs. “dormir,” and “comida” vs. “dormir” did not reach the statistical significance threshold in any scenario, which may reflect smaller linguistic contrasts and more overlapping cognitive processing. These results are consistent with previous studies on imagined speech (Datta and Boulgouris, 2021) that have grouped words according to their grammatical class (verbs and nouns), showing that it is possible to classify between these groups and that there are consistent neural patterns within each class. Additionally, based on the confusion matrices obtained in the binary classification, we observed that long words are decoded with higher accuracy. This finding aligns with the results reported by Nguyen et al. (2017), who performed a binary classification between the words *in* and *cooperate*, finding that long words are easier to classify. However, the results obtained in the multiclass classification indicate that the word “si” was the most discriminable among the others.

Given this trend, in which words with different durations were classified with higher accuracy (see Figure 6), it is considered relevant to evaluate the performance of the different classification scenarios using a time window corresponding to the duration of the shortest word (262 ms, in our case for the word “si”). Although lower performance would be expected due to the partial loss of information in longer words, this analysis would help determine whether the improvement in classification is solely due to the length of the signal or if there are informative components related to linguistic processing. However, this analysis requires further exploration and is proposed as a direction for future work.

Since the one-syllable words showed better performance compared to those with more than one syllable, the classification *short words vs. long words* was proposed. In this case, the intra-subject mixed speech training scenario achieved an improvement of 3.22% over the intra-subject *imagined speech* training scenario, with statistical significance. Furthermore, the classification results did not show a systematic bias toward either class (short or long words), indicating that the performance improvements were not driven by class imbalance but rather by genuine neural discriminability. Additionally, the trend observed in the word vs. word classification was repeated: a greater number of participants exceeded 70% accuracy in the intra-subject mixed speech training scenario, although the highest accuracy value (86.30%) was recorded in the intra-subject *imagined speech* training scenario, still by participant 22.

In the multi-class classification, three out four scenarios exceeded the statistical significance threshold (26.05%). The highest average accuracy was 31.68% in the intra-subject mixed speech training scenario, followed by 30.40% in the intra-subject *imagined speech* training scenario, however, the difference between them was not statistically significant, except when compared to the intra-subject *overt speech* training scenario. Although the overall accuracy values may appear modest, they are consistent with recent studies addressing imagined speech decoding challenges using EEG (e.g., Carvalho et al., 2024), where multiclass accuracies of ~33%–36% (for 5 and 6 classes) have been reported. These results reflect the inherent complexity of multiclass imagined speech decoding, which is limited by factors such as low signal-to-noise ratio, overlapping neural patterns, and the subtle nature of internal speech representations. Furthermore, it was observed that the highest individual accuracy (45%) was achieved in the intra-subject *imagined speech* training scenario, whereas the intra-subject mixed speech training scenario outperformed it in terms of the number of participants who exceeded both the statistical significance threshold and 40% accuracy. Together, these findings suggest that while multiclass decoding remains challenging, combining overt and imagined speech data may contribute to more robust and generalizable models across participants.

The scenario that uses only *overt speech* data to train the model and *imagined speech* data for testing, showed the lowest performance in both binary and multi-class classification. Regarding multi-subject *over* augmented mixed-speech training scenario, where *overt speech* data from all participants is mixed with a portion of *imagined speech* data from the participant to be evaluated, no improvements were observed compared to training exclusively with *imagined speech* or with mixed speech in the intra-subject configuration. Therefore, the standard method (the intra-subject *imagined speech* training) outperforms those proposed in the intra-subject *overt speech* training scenario and the multi-subject *overt* augmented mixed-speech training scenario. These results are consistent with those reported in Rekrut et al. (2022), where no significant improvements were found when using only *overt speech* data for training to classify *imagined speech*, employing CSP-based features and a random forest classifier. In Lee et al. (2023), a convolutional autoencoder was proposed to transfer *overt speech* EEG features to *imagined speech* classification, comparing it with two methods (CSP+LDA and EEGNet). No improvements were found when including *overt speech* data in those two latter methods either, achieving a 7.42% increase only when using the proposed model. One possible explanation for not finding improvements solely by using a model as complex as the one mentioned in Lee et al. (2023) could be that, in both studies, no portion of *imagined speech* data was included in the training, which may have limited the model's ability to generalize to *imagined speech* signals.

In summary, our findings suggest that combining *overt* and *imagined speech* data from the same participant for model training provides relevant and consistent features for classification, while the inter-subject approach or the exclusive use of *overt speech* did not perform as strongly with this dataset. The main limitations of this work lie in the need for more data to improve classification and the fact that acquiring *overt* and *imagined speech* without visual stimuli could provide purer speech-related signals. Nevertheless, although including *imagined speech* data in the training goes against the goal of reducing time and complexity in data acquisition, this approach offers a method to improve classification and capitalize on the early stages of the disease in patients, facilitating BCI training with intuitive tasks such as use *overt speech* before speech production is compromised.

## 5 Conclusions

These findings indicate that overt and *imagined speech* share similar neural responses within the first 500 ms, providing a reliable temporal window for classification. Moreover, combining data from overt and *imagined speech* for the same participant significantly improves *imagined speech* classification, even though the highest individual accuracy was obtained using only *imagined speech*. Nonetheless, incorporating *overt speech* data led to a greater number of participants surpassing statistical thresholds. The multi-class classification setting also benefited from mixing overt and *imagined speech* data, albeit with improvements that were not statistically significant. Expanding the dataset and removing visual stimuli during the period of interest could further refine this approach, while incorporating *imagined speech* data into training strategies may facilitate the transition to BCIs for individuals with progressive speech loss.

## Data Availability

The raw data supporting the conclusions of this article will be made available by the authors, without undue reservation.
