# Supplementary material for: From pronounced to imagined: improving speech decoding with multi-condition EEG data
Source: Front Neuroinform. 2025 Jun 27;19:1583428. doi: 10.3389/fninf.2025.1583428 (PMC12245923; doi:10.3389/fninf.2025.1583428)
Supplement: Supplementary file 1 [file Data_Sheet_1.pdf]

Supplementary Material

Table S1 shows the parameters used to train the EEGNet in the different classification scenarios. Figure S1 shows the classification accuracies per participant for the pairs of words that surpassed the threshold of statistical significance in the intra-subject scenarios 1 and 2 in word vs. word classification. The results of the two remaining pairs ('si' vs. 'comida' and 'no' vs. 'dormir') can be found in the manuscript.

Table S1. Parameters used for EEGNet training.

Case	Classification scenario	F1	D	Epochs
Word vs. Word	Intra-subject scenario 1	32	16	400
Word vs. Word	Intra-subject scenario 2	64	32	250
Word vs. Word	Intra-subject scenario 3	64	32	200
Word vs. Word	Inter-subject scenario 1	16	8	400
Multiclass	Intra-subject scenario 1	32	16	200
Multiclass	Intra-subject scenario 2	32	16	200
Multiclass	Intra-subject scenario 3	32	16	250
Multiclass	Inter-subject scenario 1	32	16	300

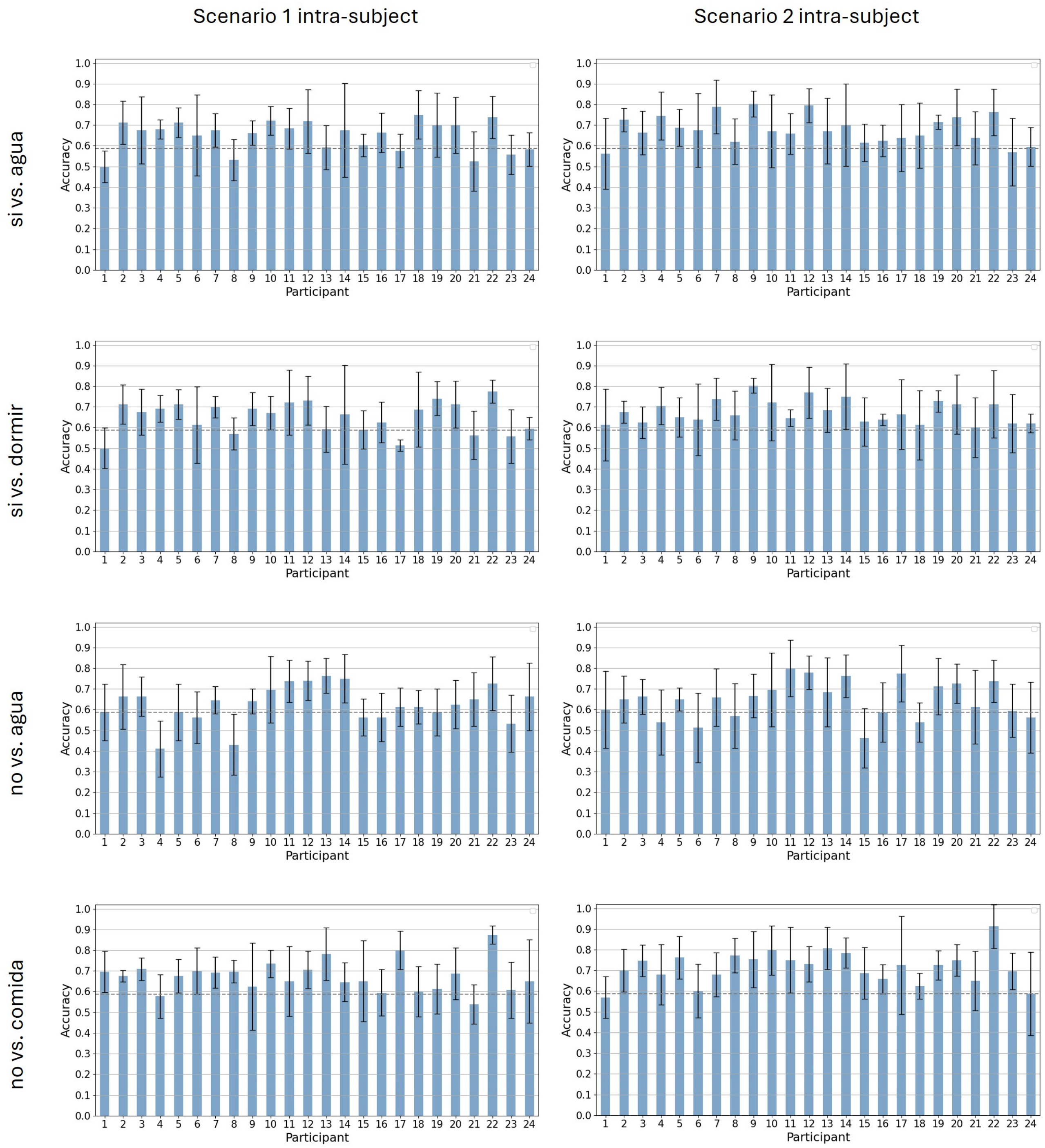

Figure S1. Classification accuracies per participant for the pairs of words that surpassed the threshold of statistical significance in the intra-subject scenarios 1 and 2 in word vs. word classification. The error bar represents the standard deviation obtained by averaging values from 5-fold cross-validation. The statistical significance threshold is indicated by the dashed line located at 58.75%.

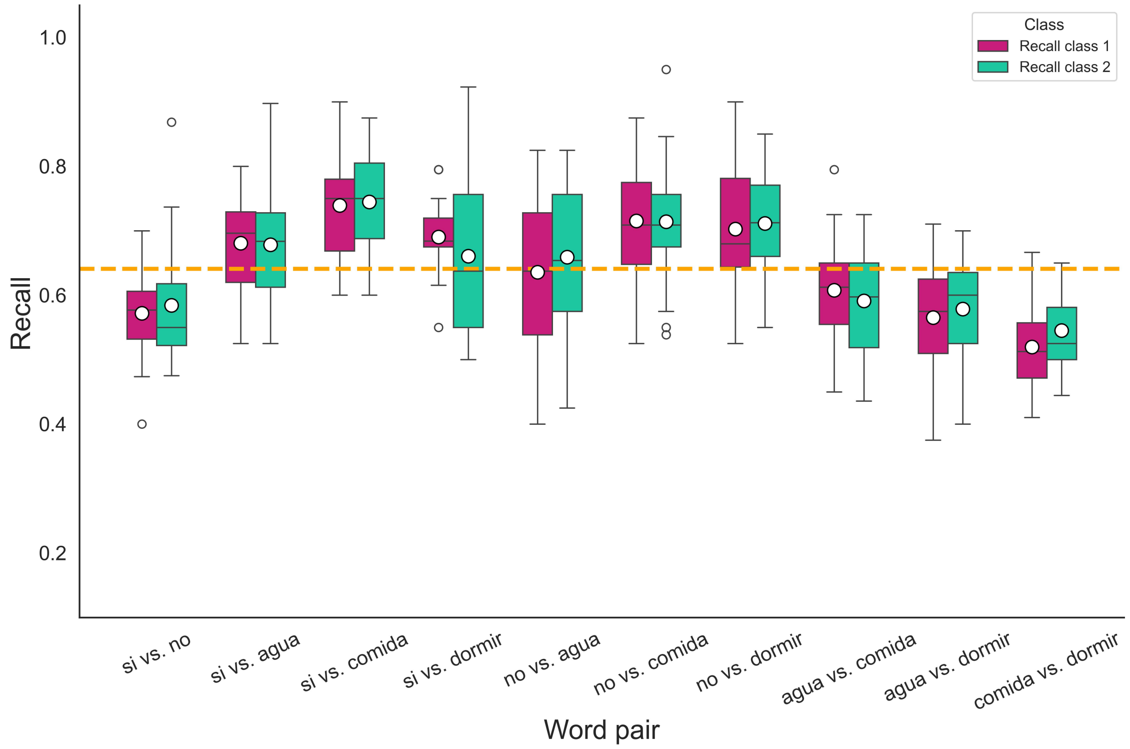

Figure S2. Comparison of the recalls for class 1 and class 2 in scenario 2 (intra-subject). The statistical significance threshold is indicated by the dashed line located at 58.75%. The white dot in the center of each boxplot represents the mean. No statistically significant differences were found between the recalls of the two classes for any of the word pairs (Wilcoxon signed-rank test, $p < 0.05$).

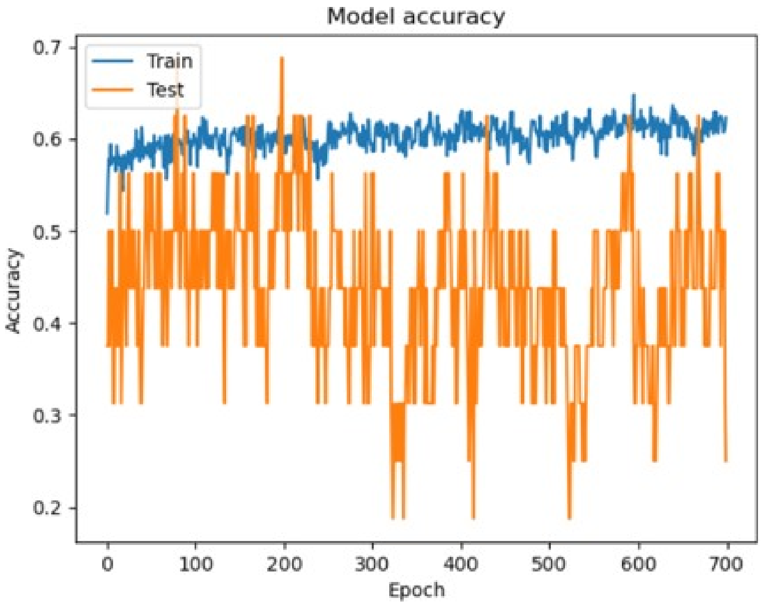

Figure S3. Model accuracy curve for the multi-subject imagined augmented training scenario. Representative accuracy curves during model training and testing for the first fold of cross-validation. In this scenario, imagined speech data from all participants was used for training, and the model was evaluated on imagined speech data from Subject 22, the best-performing participant under intra-subject conditions and using the word pair ‘si’ vs. ‘comida. Despite this, test accuracy was highly unstable and showed no clear improvement trend over the epochs.

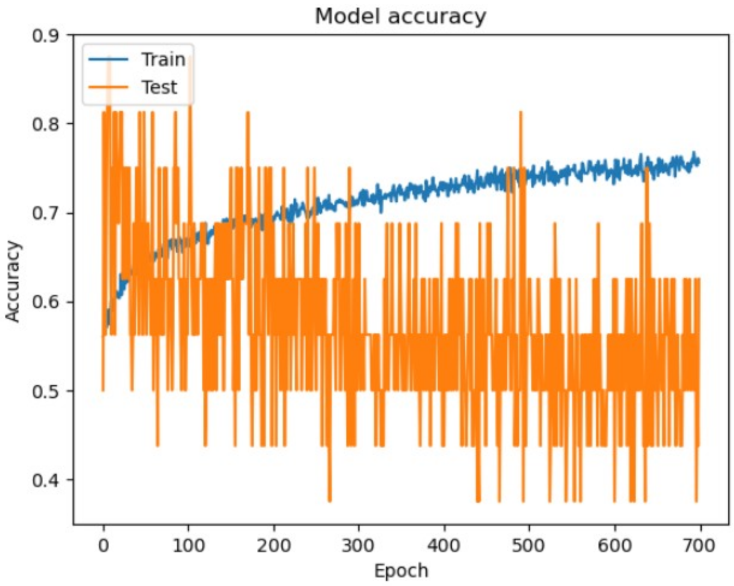

Figure S4. Model accuracy curve for the multi-subject mixed augmented training scenario. Representative accuracy curves during model training and testing for the first fold of cross-validation. In this scenario, imagined and overt speech data from all participants was used for training, and the model was evaluated on imagined speech data from Subject 22, using the word pair ‘si’ vs. ‘comida. Although a larger amount and greater diversity of data was used, the model still did not show clear convergence or stability in test accuracy.
